# Supplementary material for: Coordinated interactions between endothelial cells and macrophages in the islet microenvironment promote β cell regeneration
Source: NPJ Regen Med. 2021 Apr 6;6:22. doi: 10.1038/s41536-021-00129-z (PMC8024255; doi:10.1038/s41536-021-00129-z)
Supplement: Supplementary file 1 — Combined supplementary material [file 41536_2021_129_MOESM1_ESM.pdf]

SUPPLEMENTARY INFORMATION

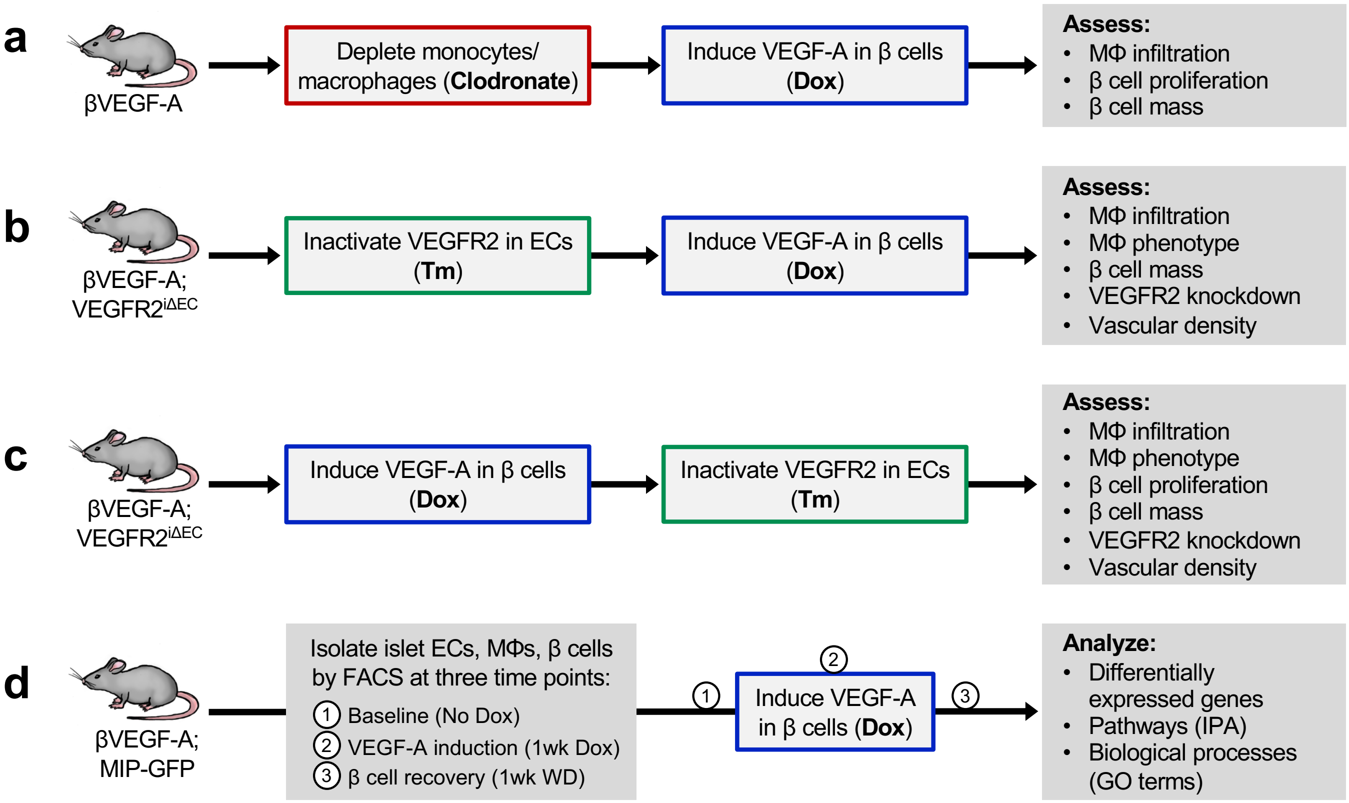

Supplementary Figure 1. Schematic representation of experiments to determine the role of macrophages and endothelial cells in the βVEGF-A mouse model. (a) Clodronate liposomes were used to suppress macrophage (MΦ) infiltration, followed by administration of doxycycline (Dox) to overexpress VEGF-A in β cells. **(b–c)** To modulate endothelial cells (ECs), an inducible EC-specific VEGFR2 knockout mouse model Cd5-CreER; VEGFR2$^{fl/fl}$ (VEGFR2$^{i\Delta EC}$)[26] was bred into the βVEGF-A line. Tamoxifen (Tm) was used to inactivate VEGFR2 in ECs either prior to **(b)** or after **(c)** VEGF-A induction to discern the role of proliferative and quiescent ECs, respectively in the β cell loss and recovery. **(d)** ECs, MΦs, and β cells were isolated from βVEGF-A mice at three time points; introduction of the MIP-GFP transgene facilitated β cell sorting. For additional details, see **Methods**.

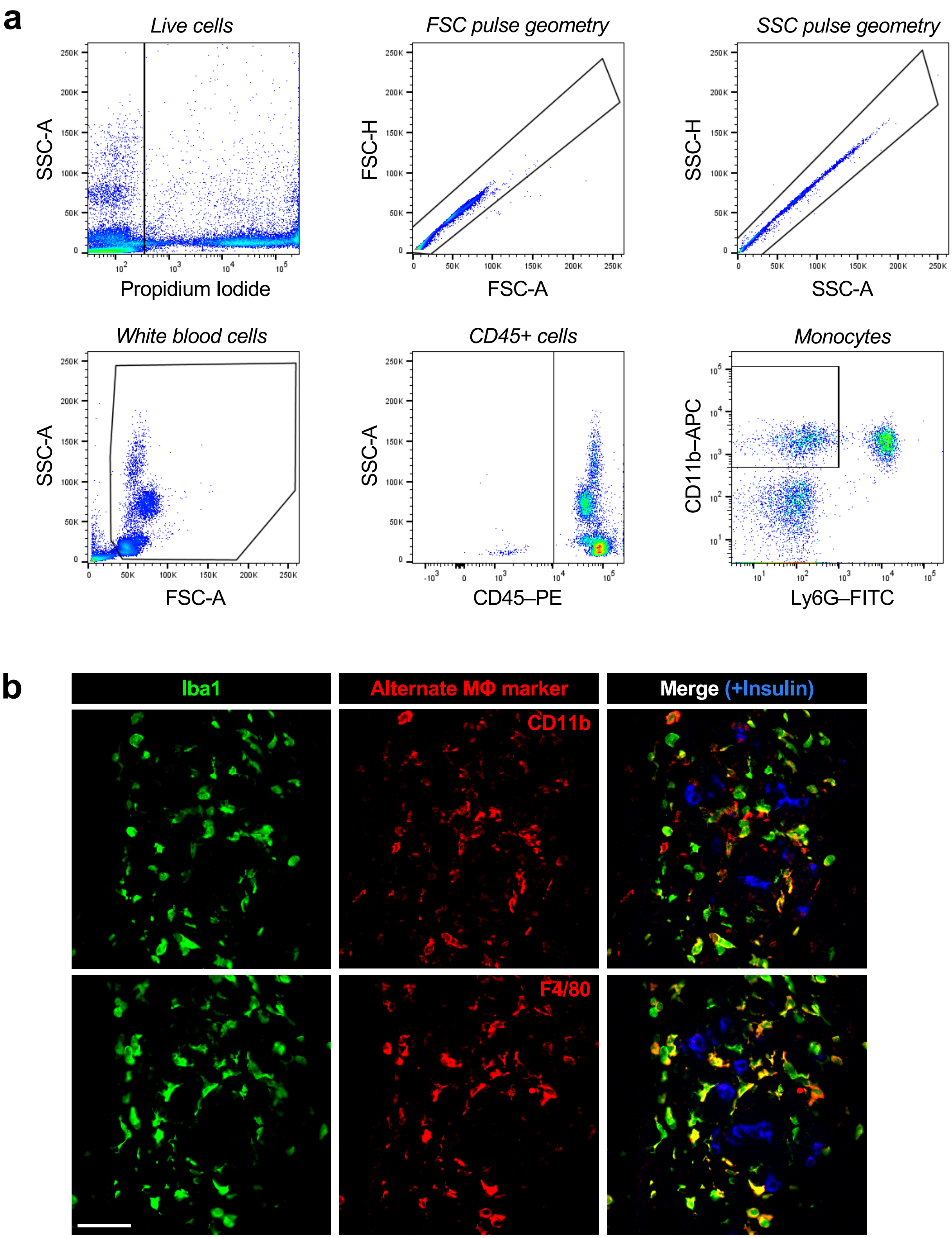

Supplementary Figure 2. Experimental design for clodronate-mediated macrophage depletion. (a) Gating strategy for flow cytometry analysis of circulating monocytes. Peripheral blood (50-100 µl) was collected from the retro-orbital sinus of βVEGF-A mice to assess monocyte depletion following clodronate treatment. Cell debris were excluded by forward scatter (FSC) and side scatter (SSC), single cells were identified by voltage pulse geometry, and non-viable cells were excluded using propidium iodide. From remaining white blood cells, CD45 was used as a pan-leukocyte marker and monocytes were identified by a $CD11b^{+}$ $Ly6G^{-}$ profile. Approximately 10,000 white blood cells were analyzed and quantified per each animal. See also **Figure 2b**. **(b)** Representative images showing islet macrophage (MΦ) infiltration during VEGF-A induction (1 wk Dox). These cells express Iba1 (green), which colocalizes with canonical MΦ markers CD11b and F4/80 (red). Scale bar, 50 µm. See also **Figure 2c**.

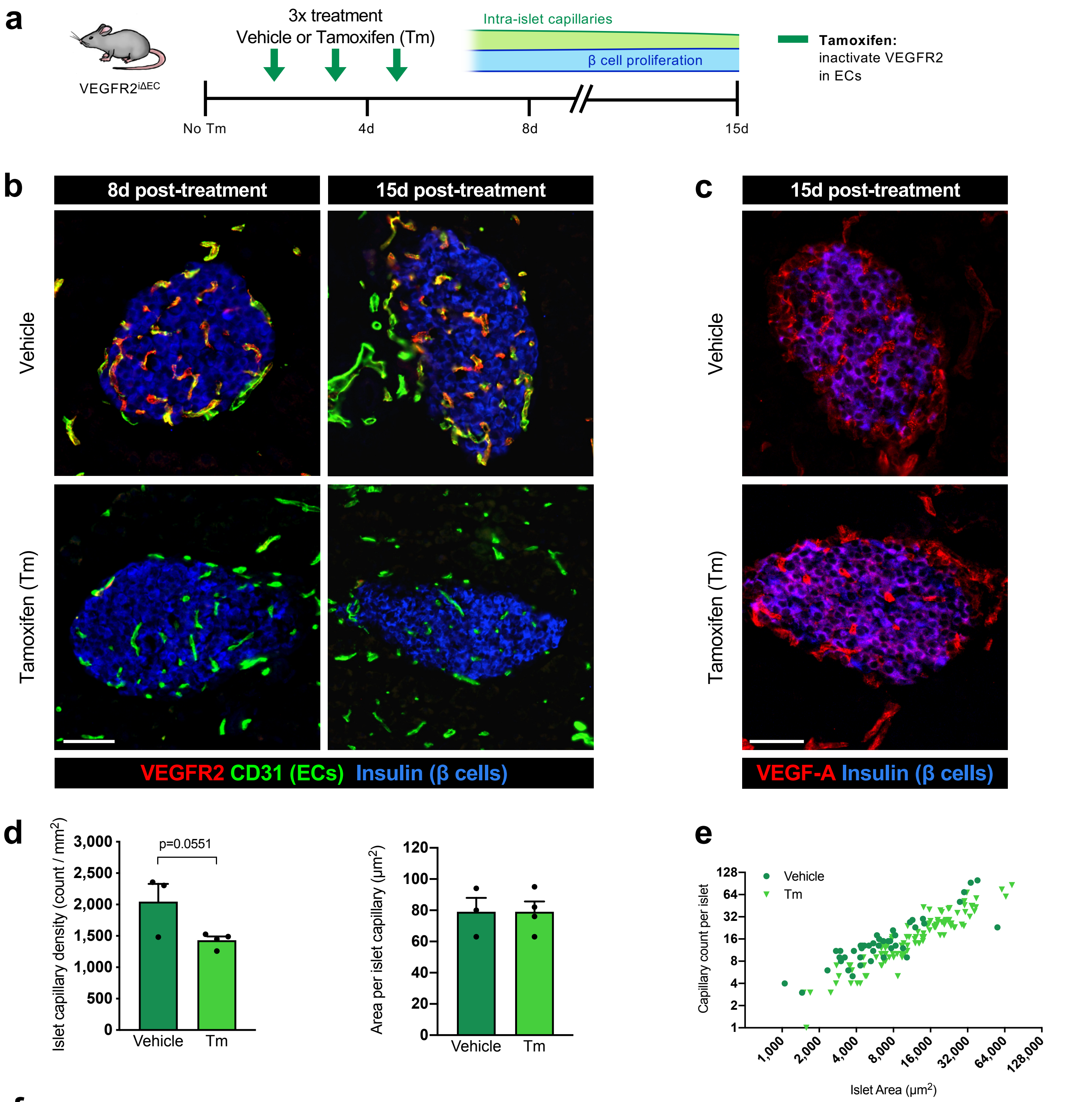

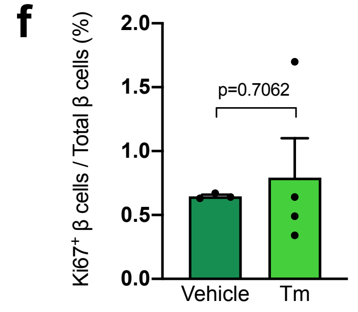

Supplementary Figure 3. Acute ablation of VEGFR2 in endothelial cells does not alter islet vascular homeostasis or β cell proliferation. (a) To inactivate VEGFR2 in endothelial cells (ECs), Cdh5-CreER^{T2}; VEGFR2$^{fl/fl}$ (VEGFR2$^{i\Delta EC}$) mice received treatments (Tx) of corn oil (vehicle) or tamoxifen (Tm; 4mg s.c.) every other day for 5 days. **(b)** Representative images of islet ECs (CD31^{+}) and β cells (Insulin^{+}) in vehicle- and Tm-treated VEGFR2$^{i\Delta EC}$ mice. VEGFR2 expression (red) is virtually undetectable in ECs at 8 or 15 days (d) after initial Tx. Scale bar, 50 μm. **(c)** VEGF-A expression (red) is maintained in β cells (blue) after VEGFR2 inactivation. Scale bar, 50 μm. **(d)** Quantification (mean + s.e.m.) of islet architecture in VEGFR2$^{i\Delta EC}$ mice at 8d post-Tx; each circle represents one animal. Islet capillary density and area per islet capillary determined by CD31^{+} and Insulin^{+} stain; 138 ± 15 x 10^5 μm^2 total islet area analyzed per animal. **(e)** In both vehicle- and Tm-treated mice, the number of islet capillaries increases proportionately with islet size. Note both axes have a base 2 logarithmic scale. **(f)** Basal β cell proliferation rate; 1,892 ± 259 cells counted per animal. Statistics shown in panels **d** and **f** reflect unpaired two-tailed t-tests.

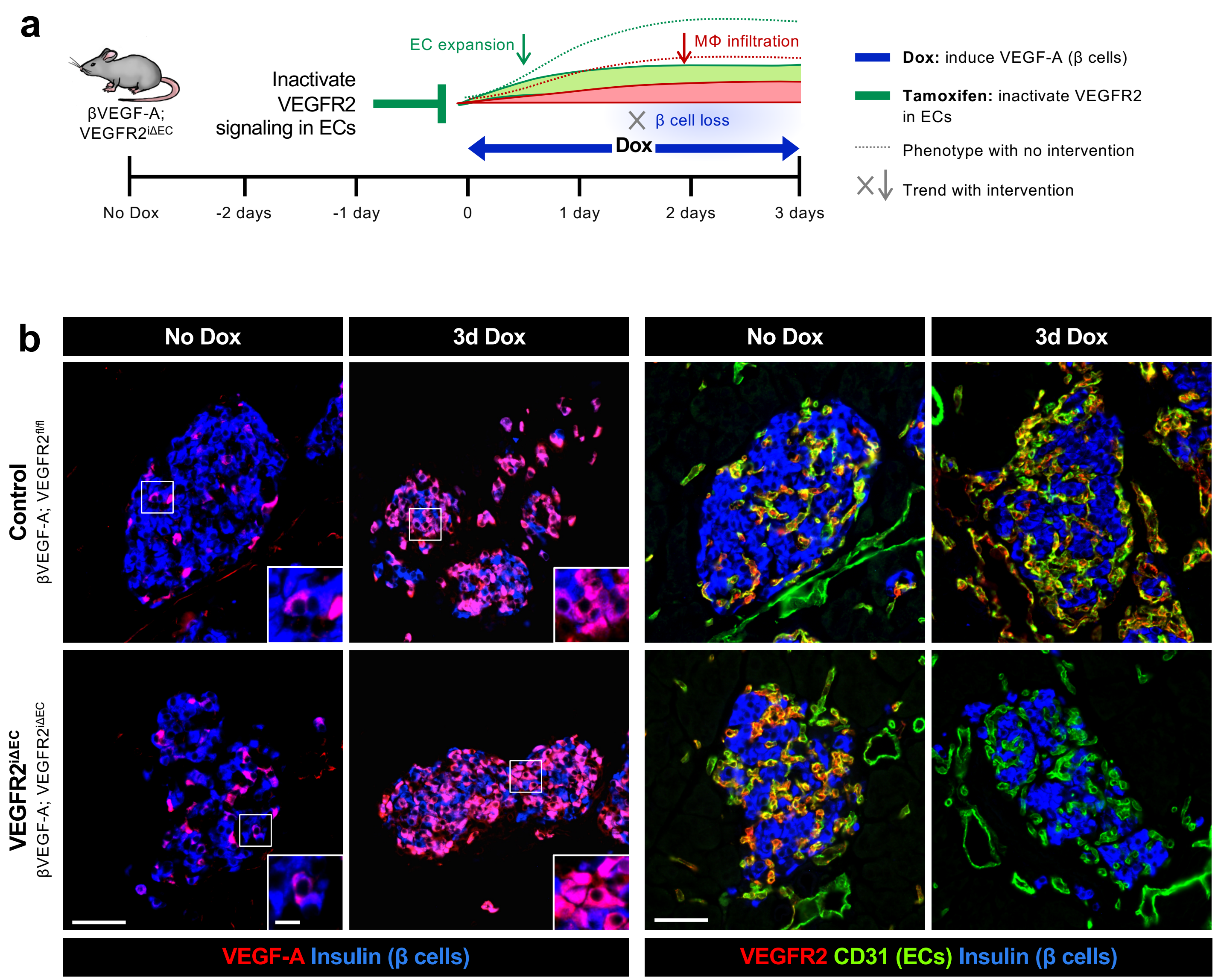

Supplementary Figure 4. Effective VEGFR2 inactivation in endothelial cells prevents islet capillary expansion by acute elevation of VEGF-A in the islet microenvironment. (a) To inactivate VEGFR2 in endothelial cells (ECs), βVEGF-A; R2$^{i\Delta EC}$ mice and βVEGF-A; R2$^{fl/fl}$ controls were treated with Tamoxifen (Tm; 4mg s.c.) prior to VEGF-A induction. **(b)** Left panel: induction of VEGFA expression at 3d Dox compared to baseline (No Dox) in βVEGF-A; R2$^{i\Delta EC}$ mice and controls; VEGF-A (red); β cells (Insulin$^+$, blue)**.** Boxes indicate regions enlarged in insets. Right panel: inactivation of VEGFR2 expression in ECs at baseline and 3d Dox; VEGFR2 (red), ECs (CD31$^+$, green); β cells (Insulin$^+$, blue)**.** Scale bars, 50 μm; inset, 10 μm.

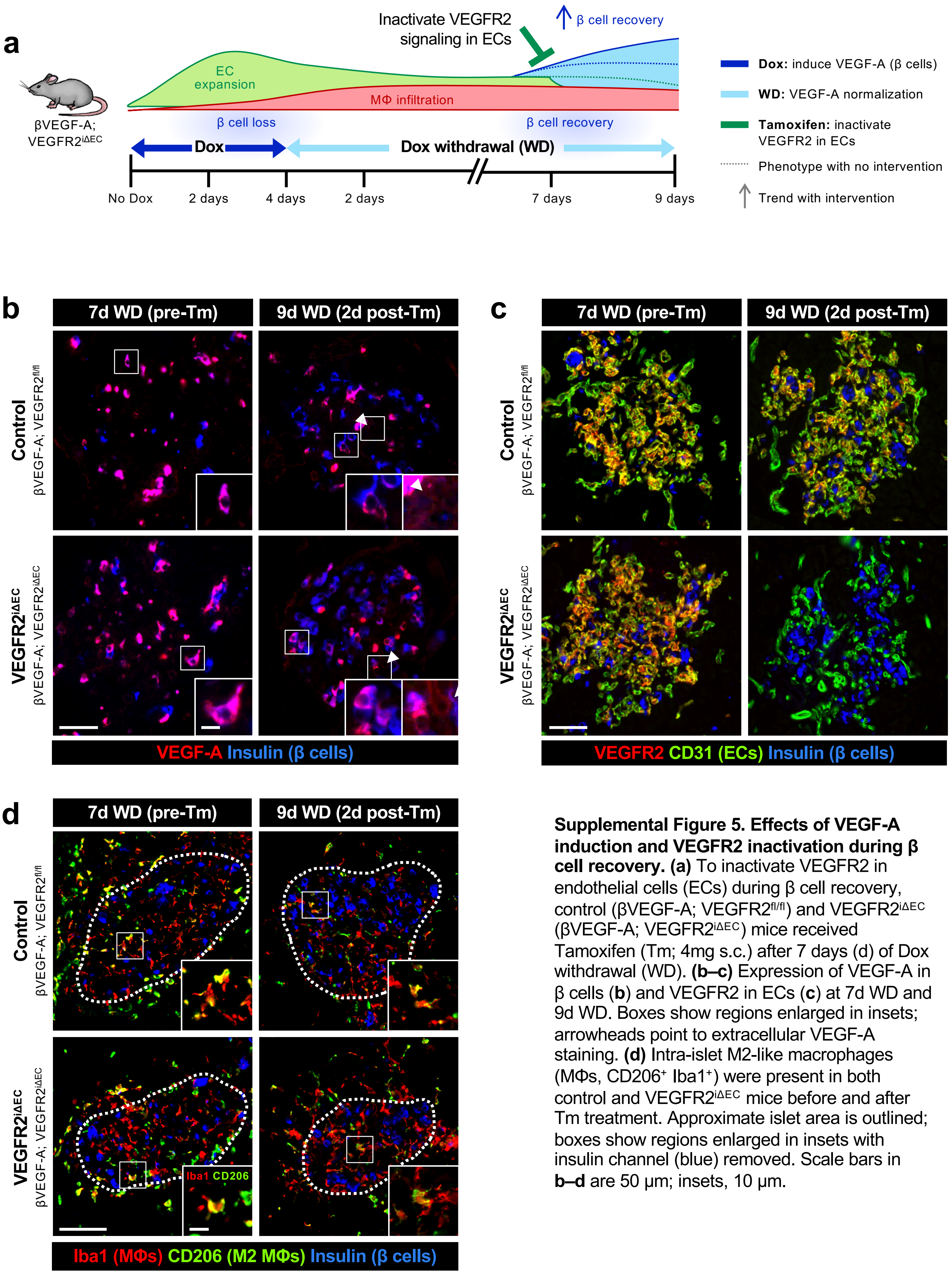

Supplemental Figure 5. Effects of VEGF-A induction and VEGFR2 inactivation during β cell recovery. (a) To inactivate VEGFR2 in endothelial cells (ECs) during β cell recovery, control (βVEGF-A; VEGFR2$^{fl/fl}$) and VEGFR2$^{i\Delta EC}$ (βVEGF-A; VEGFR2$^{i\Delta EC}$) mice received Tamoxifen (Tm; 4mg s.c.) after 7 days (d) of Dox withdrawal (WD). **(b–c)** Expression of VEGF-A in β cells (**b**) and VEGFR2 in ECs (**c**) at 7d WD and 9d WD. Boxes show regions enlarged in insets; arrowheads point to extracellular VEGF-A staining. **(d)** Intra-islet M2-like macrophages (MΦs, CD206^{+} Iba1^{+}) were present in both control and VEGFR2$^{i\Delta EC}$ mice before and after Tm treatment. Approximate islet area is outlined; boxes show regions enlarged in insets with insulin channel (blue) removed. Scale bars in **b–d** are 50 μm; insets, 10 μm.

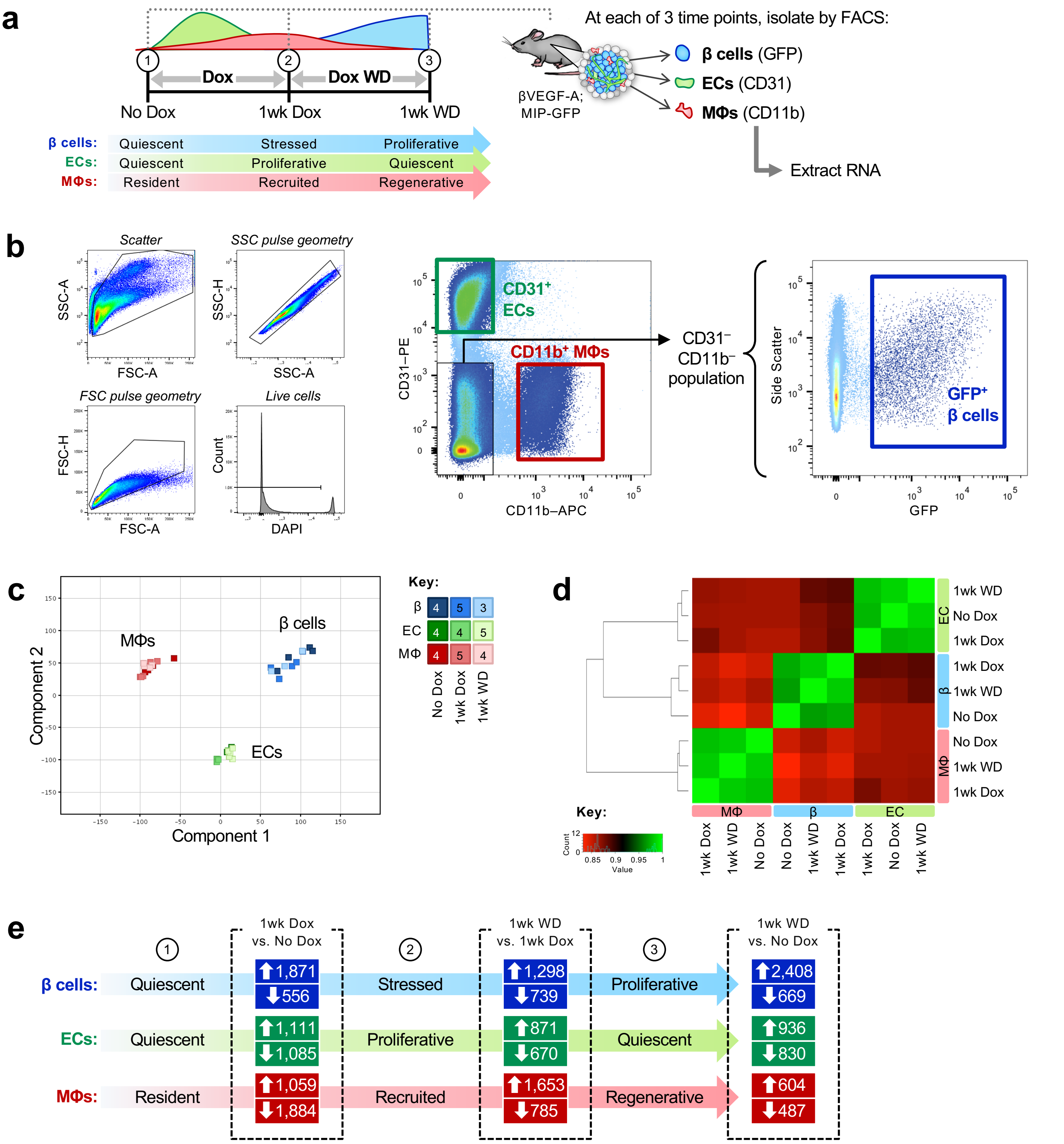

Supplemental Figure 6. Transcriptome analysis demonstrates unique gene expression profiles in β cells, endothelial cells and macrophages purified from islets during the period of β cell loss and recovery. (a–b) Dispersed islet cells from βVEGF-A; MIP-GFP mice were immunolabeled with fluorophore-conjugated anti-CD31 and anti-CD11b antibodies to separate endothelial cell (EC) and macrophage (MΦ) populations, respectively. GFP⁺ β cells were sorted from the CD31⁻ CD11b⁻ population. Initial gating is shown in first four plots. **(c)** Principal component analysis (PCA) plot shows the clustering of samples from sorted β cells (blue), ECs (green), and MΦs (red) at No Dox, 1wk Dox, and 1wk WD; n=3-5 biological replicates per time point as listed in Key. Islets from multiple mice were pooled to obtain adequate cells for each sample. **(d)** Pairwise correlation between all samples at all time points based on the Spearman correlation coefficient, which ranks and quantifies the degree of similarity between each pair of samples (perfect correlation=1; green). **(e)** Total number of genes for each cell type significantly up- or down-regulated (FC ≥2 or ≤-2) when comparing time points (dashed outlines): 1wk Dox vs. No Dox, 1wk WD vs. 1wk Dox, and 1wk WD vs. No Dox.

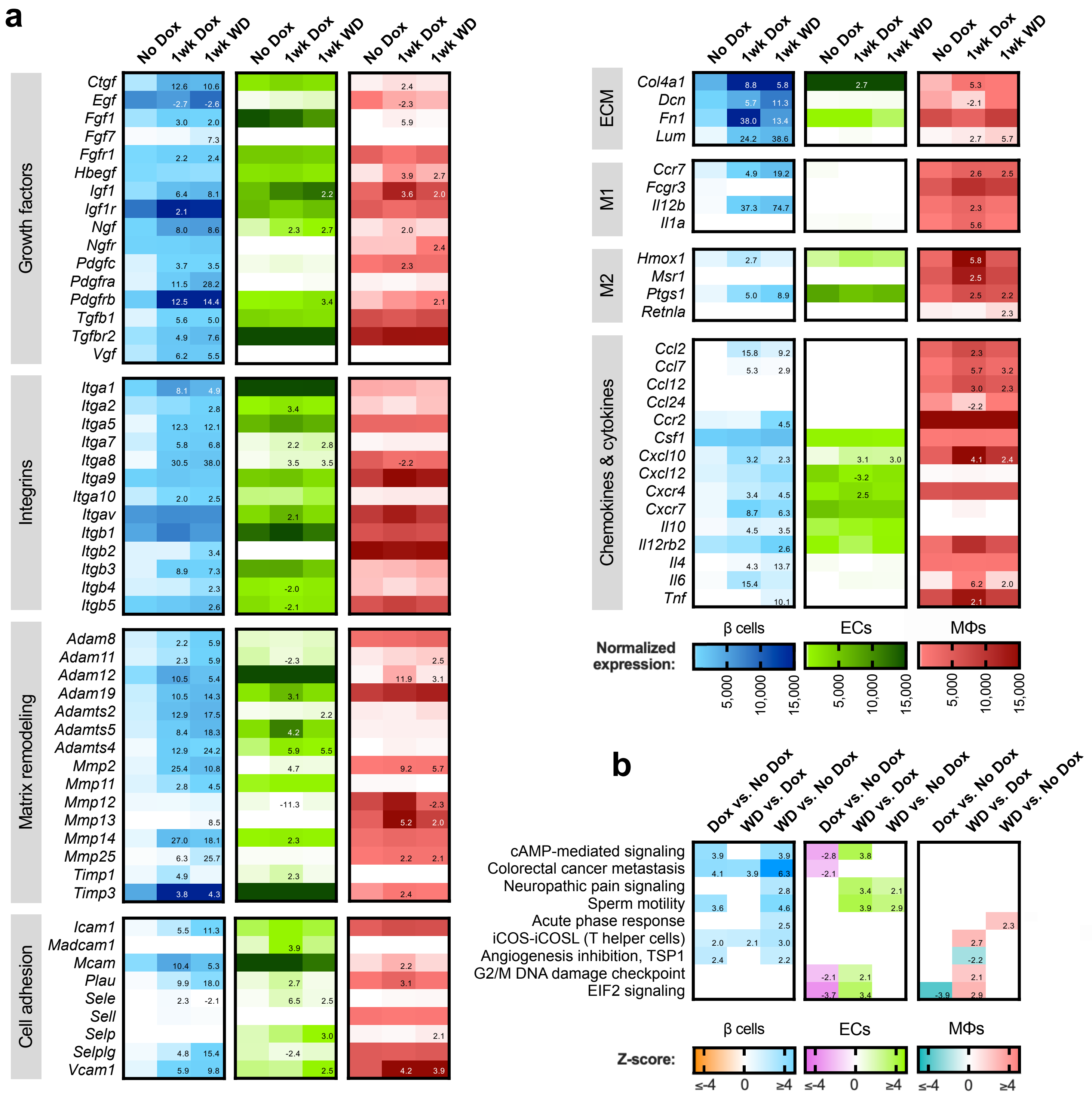

Supplemental Figure 7. Temporal changes in gene expression profiles of islet cell subpopulations by RNA-sequencing. (a) Normalized expression of selected genes in isolated β cell (blue), EC (green), and MΦ (red) samples; n=3-5 biological replicates per cell type per time point. ECM, extracellular matrix. Numbers listed on heat map in 1wk Dox and 1wk WD columns represent fold-change ≥2 or <-2 (p<0.05) as compared to No Dox. Color scales beneath each column correspond to normalized expression values ranging from 0 (white) to ≥15,000 (dark blue, green, or red for β cells, ECs, or MΦs, respectively). **(b)** Ingenuity Pathway Analysis (IPA) was applied to fold-change data (1wk Dox vs. No Dox, 1wk WD vs. 1wk Dox, and 1wk WD vs. No Dox) for β cell, EC, and MΦ populations. Pathways showing significant regulation (z-score ≥2 or ≤-2, p<0.05) in at least two cell types are shown; a full list is provided in **Supplementary Table 1**. Z-scores of relevant comparisons are listed on heat map. Color scales represent positive or negative regulation of pathways (z-score); β cell pathways are upregulated only (blue), while pathways enriched in ECs and MΦs include those positively regulated (green and pink, respectively) and those downregulated (magenta and teal, respectively).

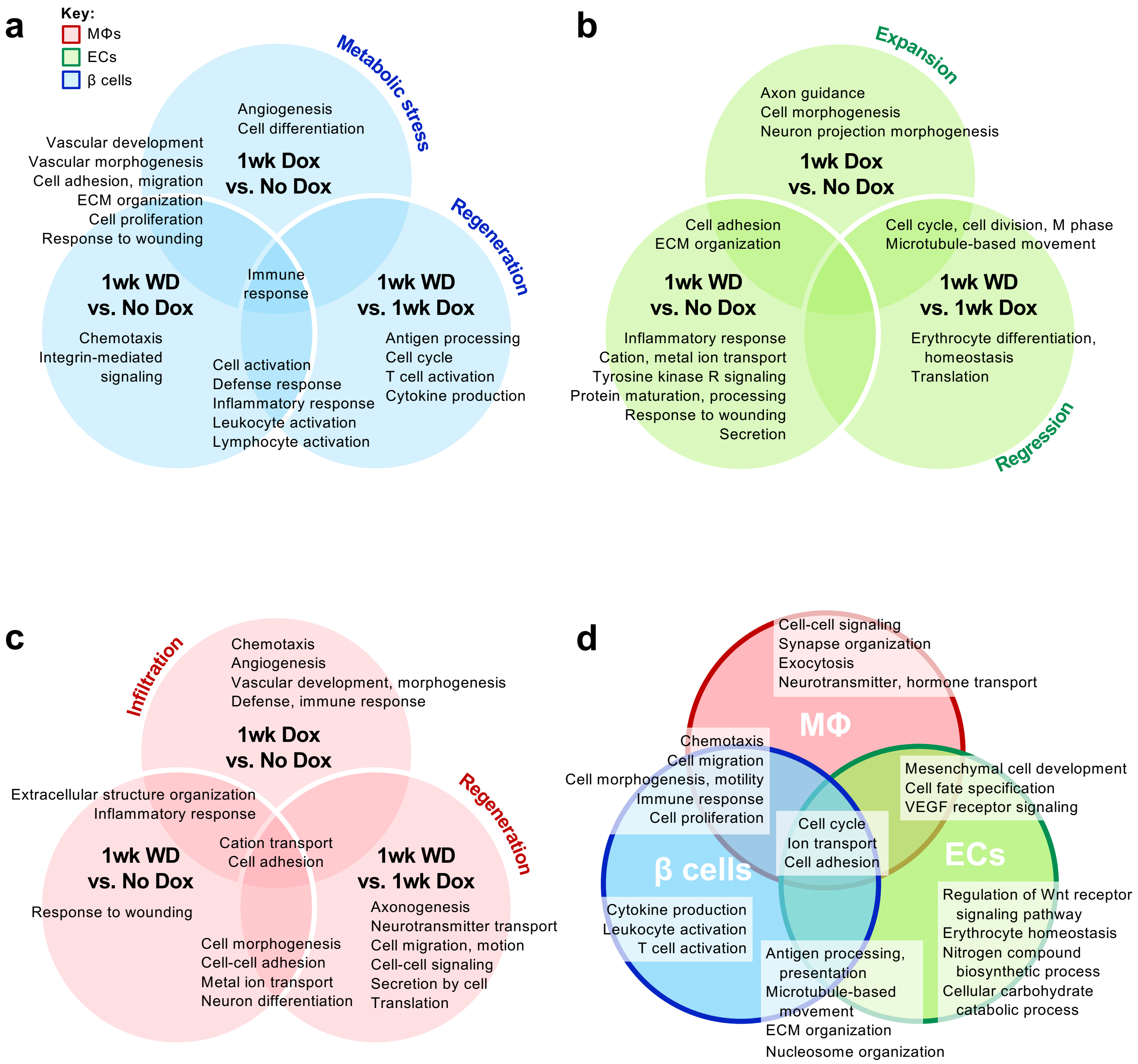

Supplemental Figure 8. Biological processes enriched in islet macrophages, endothelial cells, and β cells of βVEGF-A mice during β cell loss and recovery. Venn diagrams showing profiles of β cells **(a)**, endothelial cells (ECs) **(b)**, and macrophages (MΦs) **(c)** as determined by Gene Ontology (GO) term analysis; n=3-5 biological replicates per cell type per time point. Processes are organized by pairwise comparisons between time points (1wk Dox vs. No Dox, 1wk WD vs. 1wk Dox, and 1wk WD vs. No Dox), with location of text in Venn diagram accounting for processes that are represented in multiple time point comparisons. **(d)** Unique and shared processes of β cells, ECs, and MΦs during β cell recovery (1wk WD vs. 1wk Dox and/or 1wk WD vs. No Dox). All terms diagrammed in (**a–d**) appeared in top 20 hits (highest statistical significance) for the indicated population(s); those omitted due to space constraints are provided in **Supplementary Table 2**.

Supplementary Table 1: GO Terms for sorted cell populations

Term	Process	p value:	β cells			ECs			Macrophages		
			G1vG0	G2vG0	G2vG1	G1vG0	G2vG0	G2vG1	G1vG0	G2vG0	G2vG1
GO:0000087	M phase of mitotic cell cycle				5.31E-09	1.13E-06		2.30E-10			
GO:0000278	Mitotic cell cycle					3.29E-06		3.37E-10			
GO:0000279	M phase				8.80E-11	3.58E-07		2.60E-14			
GO:0000280	Nuclear division					3.28E-06		1.05E-09			
GO:0000902	Cell morphogenesis					6.64E-05				2.70E-09	6.23E-05
GO:0000904	Cell morphogenesis involved in differentiation					1.59E-06				2.25E-08	3.62E-04
GO:0001501	Skeletal system development		2.97E-08								
GO:0001503	Ossification						3.53E-04		0.002241		
GO:0001525	Angiogenesis		7.92E-13						6.32E-05		
GO:0001568	Blood vessel development		3.64E-21	9.44E-13					6.42E-06		
GO:0001775	Cell activation			1.79E-15	1.55E-12						
GO:0001817	Regulation of cytokine production				1.57E-10						
GO:0001944	Vasculature development		3.10E-22	1.52E-13							5.74E-06
GO:0002504	Antigen processing and presentation of peptide or polysaccharide antigen via MHC class II				5.63E-10						
GO:0002526	Acute inflammatory response						6.27E-05				
GO:0002684	Positive regulation of immune system process			1.63E-13	2.60E-13						
GO:0002694	Regulation of leukocyte activation				4.06E-09						
GO:0006323	DNA packaging							2.18E-04			
GO:0006412	Translation							1.06E-04			6.97E-06
GO:0006811	Ion transport						2.18E-04		5.92E-04		1.36E-05
GO:0006812	Cation transport						1.07E-04		4.04E-04	5.69E-07	9.98E-07
GO:0006813	Potassium ion transport						2.24E-04				3.99E-04
GO:0006836	Neurotransmitter transport										5.17E-04
GO:0006928	Cell motion			1.71E-10							4.89E-04
GO:0006935	Chemotaxis			1.78E-11					0.001387		
GO:0006952	Defense response			1.51E-14	3.64E-11				1.18E-04		
GO:0006954	Inflammatory response			2.18E-15	1.20E-09		2.59E-04		3.41E-06	4.13E-09	

Supplementary Table 1: GO Terms for sorted cell populations

		β cells			ECs			Macrophages		
**Term**	**Process** **p value:**	**G1vG0**	**G2vG0**	**G2vG1**	**G1vG0**	**G2vG0**	**G2vG1**	**G1vG0**	**G2vG0**	**G2vG1**
GO:0006955	Immune response	1.35E-10	4.67E-22	6.57E-12				1.12E-10		
GO:0007017	Microtubule-based process						4.58E-06			
GO:0007018	Microtubule-based movement				1.42E-05		8.85E-06			
GO:0007049	Cell cycle			4.50E-09	8.81E-07		4.02E-13			
GO:0007059	Chromosome segregation						1.61E-05			
GO:0007067	Mitosis				3.28E-06		1.05E-09			
GO:0007126	Meiosis						2.02E-04			
GO:0007155	Cell adhesion	1.06E-28	4.21E-36		2.29E-06	6.68E-10		4.43E-10	5.82E-14	9.81E-11
GO:0007156	Homophilic cell adhesion									2.75E-08
GO:0007167	Enzyme linked receptor protein signaling					7.97E-05				
GO:0007169	Transmembrane receptor protein tyrosine kinase signaling pathway					5.49E-05				
GO:0007229	Integrin-mediated signaling pathway		2.45E-11							
GO:0007267	Cell-cell signaling									1.22E-04
GO:0007409	Axonogenesis								2.88E-07	
GO:0007411	Axon guidance				1.55E-04					
GO:0008284	Positive regulation of cell proliferation	9.84E-11						1.03E-06		
GO:0009611	Response to wounding	6.84E-09	8.15E-18			5.42E-04		2.09E-07	3.20E-09	
GO:0015672	Monovalent inorganic cation transport					4.95E-04		8.12E-04		3.65E-06
GO:0016337	Cell-cell adhesion								1.17E-06	4.44E-06
GO:0016477	Cell migration	1.65E-08								7.82E-04
GO:0016485	Protein processing					5.36E-05				
GO:0019886	Antigen processing and presentation of exogenous peptide antigen via MHC class II			1.18E-08						
GO:0022402	Cell cycle process				1.05E-06		2.12E-13			
GO:0022403	Cell cycle phase			2.53E-09	1.90E-07		5.16E-15			
GO:0022610	Biological adhesion	1.32E-28	5.59E-36		2.52E-06	7.21E-10		4.92E-10	6.46E-14	1.07E-10
GO:0030001	Metal ion transport					2.34E-05			1.93E-08	2.25E-06

Supplementary Table 1: GO Terms for sorted cell populations

		β cells			ECs			Macrophages		
Term	Process p value:	G1vG0	G2vG0	G2vG1	G1vG0	G2vG0	G2vG1	G1vG0	G2vG0	G2vG1
GO:0030030	Cell projection organization								9.33E-07	
GO:0030155	Regulation of cell adhesion	5.67E-10								
GO:0030182	Neuron differentiation								3.32E-08	9.99E-04
GO:0030198	Extracellular matrix organization	1.11E-13	1.62E-11			6.79E-04				
GO:0030218	Erythrocyte differentiation						9.14E-04			
GO:0031175	Neuron projection development								1.48E-07	
GO:0032940	Secretion by cell					2.48E-04				8.57E-04
GO:0032989	Cellular component morphogenesis				1.22E-04				3.34E-08	6.10E-04
GO:0032990	Cell part morphogenesis								2.09E-07	
GO:0034101	Erythrocyte homeostasis						5.06E-04			
GO:0035295	Tube development	7.39E-09								
GO:0042127	Regulation of cell proliferation	1.27E-14	7.62E-13						8.49E-07	
GO:0042330	Taxis			1.78E-11				0.001387		
GO:0043062	Extracellular structure organization	2.74E-10	5.05E-11		1.18E-04			0.001091	1.38E-06	
GO:0044057	Regulation of system process					7.29E-04				
GO:0045321	Leukocyte activation		4.48E-15	3.83E-12						
GO:0045597	Positive regulation of cell differentiation	2.62E-08								
GO:0045765	Regulation of angiogenesis	3.60E-08								
GO:0045785	Positive regulation of cell adhesion	1.37E-08								
GO:0046649	Lymphocyte activation		3.34E-12	3.00E-10						
GO:0046903	Secretion					7.69E-05				
GO:0048285	Organelle fission				9.66E-06		3.91E-09			
GO:0048514	Blood vessel morphogenesis	7.41E-20	2.09E-12					4.47E-05		
GO:0048666	Neuron development								8.58E-09	
GO:0048667	Cell morphogenesis involved in neuron differentiation				5.62E-05				1.10E-08	
GO:0048812	Neuron projection morphogenesis				2.34E-04				1.70E-07	
GO:0048858	Cell projection morphogenesis								5.79E-08	7.17E-04

Supplementary Table 1: GO Terms for sorted cell populations

		β cells			ECs			Macrophages		
**Term**	**Process** **p value:**	**G1vG0**	**G2vG0**	**G2vG1**	**G1vG0**	**G2vG0**	**G2vG1**	**G1vG0**	**G2vG0**	**G2vG1**
GO:0050863	Regulation of T cell activation			6.50E-09						
GO:0050865	Regulation of cell activation			1.74E-09						
GO:0050867	Positive regulation of cell activation			4.69E-09						
GO:0050870	Positive regulation of T cell activation			4.71E-11						
GO:0051094	Positive regulation of developmental process	3.48E-09								
GO:0051249	Regulation of lymphocyte activation			6.63E-09						
GO:0051301	Cell division				5.30E-06		8.55E-10			
GO:0051321	Meiotic cell cycle						2.82E-04			
GO:0051327	M phase of meiotic cell cycle						2.02E-04			
GO:0051604	Protein maturation					1.15E-04				
GO:0051605	Protein maturation by peptide bond cleavage					4.52E-06				
GO:0060348	Bone development							0.001779		

Supplementary Table 2: Mouse strains utilized in experimental models

Abbreviation	MGI Nomenclature or Strain Name	References
Cd5-CreER	Tg(Cdh5-cre/ERT2)#Ykub	Okabe *et al.*, 2014[24]
MIP-GFP	B6.Cg-Tg(Ins1-EGFP)1Hara/J	Hara et al., 2003[83]
RIP-rtTA	Tg(Ins2-rtTA)2Efr	Milo-Landesman *et al.*, 2001[80]
TetO-VEGF	n/a	Efrat *et al.*, 1995[79]; Ohno-Matsui *et al.*, 2002[81]
$VEGFR2^{fl/fl}$	$Kdr^{tm2Sato}$	Hooper *et al.*, 2009[82]

Supplementary Table 3: Breeding scheme to generate βVEGF-A; $VEGFR2^{i\Delta EC}$ mice

Cross	Male breeder	Female breeder	Desired Offspring	Frequency
A1	$Cd5\text{-}CreER^{tg/+}$	$VEGFR2^{fl/fl}$	$Cd5\text{-}CreER^{tg/+}$; $VEGFR2^{fl/+}$	*50%*
A2	$Cd5\text{-}CreER^{tg/+}$; $VEGFR2^{fl/+}$	$VEGFR2^{fl/fl}$	$Cd5\text{-}CreER^{tg/+}$; $VEGFR2^{fl/fl}$	*25%*
A3	$Cd5\text{-}CreER^{tg/+}$; $VEGFR2^{fl/fl}$	$RIP\text{-}rtTA^{tg/tg}$	$Cd5\text{-}CreER^{tg/+}$; $VEGFR2^{fl/+}$; $RIP\text{-}rtTA^{tg/+}$	*50%*
A4	$Cd5\text{-}CreER^{tg/+}$; $VEGFR2^{fl/+}$; $RIP\text{-}rtTA^{tg/+}$	$VEGFR2^{fl/fl}$	$Cd5\text{-}CreER^{tg/+}$; $VEGFR2^{fl/fl}$; $RIP\text{-}rtTA^{tg/+}$	*12.5%*
B1	$VEGFR2^{fl/fl}$	$TetO\text{-}VEGFA^{tg/tg}$	$TetO\text{-}VEGFA^{tg/+}$; $VEGFR2^{fl/+}$	*100%*
B2	$TetO\text{-}VEGFA^{tg/+}$; $VEGFR2^{fl/+}$	$TetO\text{-}VEGFA^{tg/tg}$	$TetO\text{-}VEGFA^{tg/tg}$; $VEGFR2^{fl/+}$	*50%*
B3	$TetO\text{-}VEGFA^{tg/+}$; $VEGFR2^{fl/+}$	$TetO\text{-}VEGFA^{tg/tg}$; $VEGFR2^{fl/+}$	$TetO\text{-}VEGFA^{tg/tg}$; $VEGFR2^{fl/fl}$	*25%*
C1	$Cd5\text{-}CreER^{tg/+}$; $VEGFR2^{fl/fl}$; $RIP\text{-}rtTA^{tg/+}$	$TetO\text{-}VEGFA^{tg/tg}$; $VEGFR2^{fl/fl}$	$TetO\text{-}VEGFA^{tg/+}$; $Cd5\text{-}CreER^{tg/+}$; $VEGFR2^{fl/fl}$; $RIP\text{-}rtTA^{tg/+}$	*25%*

Supplementary Table 4: PCR primers and conditions for genotyping

Gentoype	Primers	PCR Conditions	Product(s)
Cd5-CreER	5′- GCG GTC TGG CAG TAA AAA CTA TC -3′<br>3′- TT CAC TGT CGT TAC GAC AAA GTG -5′	93°C 3’<br>---------------------------<br>93°C 20”<br>60°C 20” 30 cycles<br>72°C 45”<br>---------------------------<br>72°C 5’<br>4°C hold	100 bp
MIP-GFP	5′- AAG TTC ATC TGC ACC ACC G -3′ (GFP)<br>3′- GC GTG GTA GAA GAA GTT CCT -5′ (GFP)<br><br>5′- CTA GGC CAC AGA ATT GAA AGA TCT -3′ (IC)<br>3′- C CTA CTA CGA TCT TAA AGG TGG ATG -5′ (IC)	94°C 1.5’<br>---------------------------<br>94°C 30”<br>60°C 1’ 35 cycles<br>72°C 1’<br>---------------------------<br>72°C 2’<br>4°C hold	173 bp (*GFP*)<br>324 bp (IC)
RIP-rtTA	5′- GTG AAG TGG GTC CGC GTA CAG -3′<br>3′- CTA CGG GAA CCT TAA CTG CTC ATG -5′	92°C 2’<br>---------------------------<br>94°C 30”<br>57°C 30” 30 cycles<br>72°C 30”<br>---------------------------<br>72°C 10’<br>4°C hold	400 bp
TetO-VEGF	5′- TCG AGT AGG CGT GTA CGG -3′<br>3′- GCT ACG CCC CCG ACG ACG -5′	95°C 4’<br>---------------------------<br>95°C 1’<br>57°C 30” 29 cycles<br>72°C 1’<br>---------------------------<br>72°C 10’<br>4°C hold	420 bp
VEGFR2$^{fl/fl}$	5′ - CCA CAG AAC AAC TCA GGG CTA - 3′<br>3′- AA AGG TCT CTG AAA CGA GGG -5′	94°C 2’<br>---------------------------<br>94°C 20”<br>65°C 15” 10 cycles<br>68°C 10”<br>---------------------------<br>94°C 15”<br>60°C 15” 28 cycles<br>72°C 10”<br>---------------------------<br>72°C 1’<br>4°C hold	179 bp (*VEGFR2*)<br>230 bp (*VEGFR2^{loxP}*)

GFP, green fluorescent protein; IC, internal control

Supplementary Table 5: Antibodies used for immunohistochemistry and flow cytometry

Antigen/Conjugate	Species	Source	Catalog #	Application	Dilution
Caveolin-1	Rabbit	Abcam	ab2910	IHC (1º)	1:2000
CD11b	Rat	Novus	NB600-1327	IHC (1º)	1:400
CD11b – APC	Rat	BD Pharmingen	561690	FC	1:100, 1:500
CD206 (Mrc1)	Rat	BioLegend	141701	IHC (1º)	1:500
CD31	Rat	BD Pharmingen	553370	IHC (1º)	1:100
CD31 – PE	Rat	BD Pharmingen	561073	FC	1:500
CD45 – PE	Rat	BD Pharmingen	561087	FC	1:500
Col-IV	Rabbit	Abcam	ab6586	IHC (1º)	1:1000
F4/80	Rat	ThermoFisher Scientific	14-4801-82	IHC (1º)	1:200
Goat IgG – Cy3	Donkey	Jackson Immunoresearch	705-165-147	IHC (2º)	1:500
Guinea pig IgG – Cy2	Donkey	Jackson Immunoresearch	706-225-148	IHC (2º)	1:500
Guinea pig IgG – Cy5	Donkey	Jackson Immunoresearch	706-175-148	IHC (2º)	1:200
Iba1	Rabbit	Wako	019-19741	IHC (1º)	1:500
Iba1	Rabbit	Abcam	ab221790	IHC (1º)	1:1000
Insulin	Guinea pig	Dako	A0564	IHC (1º)	1:1000
Insulin	Guinea pig	Cell Marque	273A-15	IHC (1º)	1:500
Ki67	Rabbit	Abcam	ab15580	IHC (1º)	1:5000
Ly6G-FITC	Rat	BD Pharmingen	551460	FC	1:500
pERK	Rabbit	Cell Signaling	4370	IHC (1º)	1:200
Rabbit IgG – Cy2	Donkey	Jackson Immunoresearch	711-225-152	IHC (2º)	1:500
Rabbit IgG – Cy3	Donkey	Jackson Immunoresearch	711-165-152	IHC (2º)	1:500
Rat IgG – Cy2	Donkey	Jackson Immunoresearch	712-225-153	IHC (2º)	1:500
VEGF-A	Goat	R&D Systems	AF564	IHC (1º)	1:200
VEGFR2	Goat	R&D Systems	AF644	IHC (1º)	1:2000

1º, primary antibody; 2º, secondary antibody; FC, flow cytometry; IHC, immunohistochemistry
