# Supplementary material for: Coordinated interactions between endothelial cells and macrophages in the islet microenvironment promote β cell regeneration
Source: NPJ Regen Med. 2021 Apr 6;6:22. doi: 10.1038/s41536-021-00129-z (PMC8024255; doi:10.1038/s41536-021-00129-z)
Supplement: Supplementary file 2 — Reporting Summary Checklist [file 41536_2021_129_MOESM2_ESM.pdf]

nature research

Corresponding author(s): Brissova, Marcela

Last updated by author(s): February 9, 2021

Reporting Summary

Nature Research wishes to improve the reproducibility of the work that we publish. This form provides structure for consistency and transparency in reporting. For further information on Nature Research policies, see our Editorial Policies and the Editorial Policy Checklist.

Statistics

For all statistical analyses, confirm that the following items are present in the figure legend, table legend, main text, or Methods section.

n/a	Confirmed	
☐	☒	The exact sample size (*n*) for each experimental group/condition, given as a discrete number and unit of measurement
☐	☒	A statement on whether measurements were taken from distinct samples or whether the same sample was measured repeatedly
☐	☒	The statistical test(s) used AND whether they are one- or two-sided<br>*Only common tests should be described solely by name; describe more complex techniques in the Methods section.*
☒	☐	A description of all covariates tested
☐	☒	A description of any assumptions or corrections, such as tests of normality and adjustment for multiple comparisons
☐	☒	A full description of the statistical parameters including central tendency (e.g. means) or other basic estimates (e.g. regression coefficient) AND variation (e.g. standard deviation) or associated estimates of uncertainty (e.g. confidence intervals)
☐	☒	For null hypothesis testing, the test statistic (e.g. *F*, *t*, *r*) with confidence intervals, effect sizes, degrees of freedom and *P* value noted<br>*Give P values as exact values whenever suitable.*
☒	☐	For Bayesian analysis, information on the choice of priors and Markov chain Monte Carlo settings
☒	☐	For hierarchical and complex designs, identification of the appropriate level for tests and full reporting of outcomes
☒	☐	Estimates of effect sizes (e.g. Cohen's *d*, Pearson's *r*), indicating how they were calculated

Our web collection on statistics for biologists contains articles on many of the points above.

Software and code

Policy information about availability of computer code

Data collection	None
Data analysis	None

For manuscripts utilizing custom algorithms or software that are central to the research but not yet described in published literature, software must be made available to editors and reviewers. We strongly encourage code deposition in a community repository (e.g. GitHub). See the Nature Research guidelines for submitting code & software for further information.

Data

Policy information about availability of data

All manuscripts must include a data availability statement. This statement should provide the following information, where applicable:

- Accession codes, unique identifiers, or web links for publicly available datasets
- A list of figures that have associated raw data
- A description of any restrictions on data availability

RNA sequencing data has been deposited to the Gene Expression Omnibus (GEO) database of the National Center for Biotechnology Information (NCBI) under accession numbers GSE72546 and GSE163825. Additional datasets generated during the current study are available from the corresponding author on reasonable request.

Field-specific reporting

Please select the one below that is the best fit for your research. If you are not sure, read the appropriate sections before making your selection.

☒ Life sciences ☐ Behavioural & social sciences ☐ Ecological, evolutionary & environmental sciences

For a reference copy of the document with all sections, see nature.com/documents/nr-reporting-summary-flat.pdf

Life sciences study design

All studies must disclose on these points even when the disclosure is negative.

Sample size	Sample sizes were determined by previous studies utilizing this model (Brissova et al., Cell Metabolism 2014).
Data exclusions	We excluded any animals in which the VEGF-A transgene was not induced via Doxycycline, as evaluated by immunohistochemistry.
Replication	All experimental groups contained multiple replicates.
Randomization	For VEGFR2iΔEC experiments, animals were randomly assigned to study groups. For all other experiments, animals were genotyped for the Cre transgene and assigned to study groups accordingly (as explained in manuscript text).
Blinding	An automated algorithm was used for immunohistochemistry analysis to avoid experimenter bias.

Reporting for specific materials, systems and methods

We require information from authors about some types of materials, experimental systems and methods used in many studies. Here, indicate whether each material, system or method listed is relevant to your study. If you are not sure if a list item applies to your research, read the appropriate section before selecting a response.

Materials & experimental systems

n/a	Involved in the study
☐	☒ Antibodies
☒	☐ Eukaryotic cell lines
☒	☐ Palaeontology and archaeology
☐	☒ Animals and other organisms
☒	☐ Human research participants
☒	☐ Clinical data
☒	☐ Dual use research of concern

Methods

n/a	Involved in the study
☒	☐ ChIP-seq
☐	☒ Flow cytometry
☒	☐ MRI-based neuroimaging

Antibodies

Antibodies used	All antibodies used in this study are detailed in Supplementary Table 5.
Validation	All antibodies used in this study are commercially available and validated (see Supplementary Table 5 for manufacturer information).

Animals and other organisms

Policy information about studies involving animals; ARRIVE guidelines recommended for reporting animal research

Laboratory animals	Mus musculus; all strains used in this study are detailed in Supplementary Table 2. Due to the inefficient induction of VEGF-A in female mice (the single copy of the TetO transgene is subject to X chromosome inactivation), only male mice were used for experiments. All mice used in the study were 8-16 weeks old at the onset of experimental procedures.
Wild animals	Study did not involve wild animals.
Field-collected samples	Study did not involve samples collected from the field.
Ethics oversight	Ethical approval and guidance was provided by the Institutional Animal Care and Use Committee at Vanderbilt University Medical Center.

Note that full information on the approval of the study protocol must also be provided in the manuscript.

Flow Cytometry

Plots

Confirm that:

☒ The axis labels state the marker and fluorochrome used (e.g. CD4-FITC).

☒ The axis scales are clearly visible. Include numbers along axes only for bottom left plot of group (a 'group' is an analysis of identical markers).

☒ All plots are contour plots with outliers or pseudocolor plots.

☒ A numerical value for number of cells or percentage (with statistics) is provided.

Methodology

Sample preparation	Peripheral blood was incubated in erythrocyte lysis buffer, and isolated islets were dispersed with Accutase. Both protocols are detailed in Methods.
Instrument	LSRFortessa cell analyzer (BD Biosciences) for flow cytometry; FACSAria III cell sorter (BD Biosciences) for FACS
Software	FlowJo 7.6.5-10.71 (FlowJo LLC)
Cell population abundance	Cell purity was assessed by RNA-sequencing.
Gating strategy	Samples were assessed by scatter, SSC and FSC pulse geometry, viability, and then positivity for cell-specific markers (see Supplementary Figures 2a and 6b).

☒ Tick this box to confirm that a figure exemplifying the gating strategy is provided in the Supplementary Information.
